# Motor neuron derivation from human embryonic and induced pluripotent stem cells: experimental approaches and clinical perspectives

**DOI:** 10.1186/scrt476

**Published:** 2014-07-14

**Authors:** Irene Faravelli, Monica Bucchia, Paola Rinchetti, Monica Nizzardo, Chiara Simone, Emanuele Frattini, Stefania Corti

**Affiliations:** 1 Dino Ferrari Centre, Neuroscience Section, Department of Pathophysiology and Transplantation, University of Milan, Neurology Unit, IRCCS Foundation Ca’Granda Ospedale Maggiore Policlinico, via Francesco Sforza 35, Milan 20122, Italy

## Abstract

Motor neurons are cells located in specific areas of the central nervous system, such as brain cortex (upper motor neurons), brain stem, and spinal cord (lower motor neurons), which maintain control over voluntary actions. Motor neurons are affected primarily by a wide spectrum of neurological disorders, generally indicated as motor neuron diseases (MNDs): these disorders share symptoms related to muscular atrophy and paralysis leading to death. No effective treatments are currently available. Stem cell-derived motor neurons represent a promising research tool in disease modeling, drug screening, and development of therapeutic approaches for MNDs and spinal cord injuries. Directed differentiation of human pluripotent stem cells - human embryonic stem cells (hESCs) and human induced pluripotent stem cells (hiPSCs) - toward specific lineages is the first crucial step in order to extensively employ these cells in early human development investigation and potential clinical applications. Induced pluripotent stem cells (iPSCs) can be generated from patients’ own somatic cells (for example, fibroblasts) by reprogramming them with specific factors. They can be considered embryonic stem cell-like cells, which express stem cell markers and have the ability to give rise to all three germ layers, bypassing the ethical concerns. Thus, hiPSCs constitute an appealing alternative source of motor neurons. These motor neurons might be a great research tool, creating a model for investigating the cellular and molecular interactions underlying early human brain development and pathologies during neurodegeneration. Patient-specific iPSCs may also provide the premises for autologous cell replacement therapies without related risks of immune rejection. Here, we review the most recent reported methods by which hESCs or iPSCs can be differentiated toward functional motor neurons with an overview on the potential clinical applications.

## Introduction

Motor neurons (MNs) are differentiated cells that control voluntary actions and are affected primarily by a wide spectrum of neurological disorders, generally indicated as motor neuron diseases (MNDs). MNDs may present with a range of symptoms deriving from muscular weakness/atrophy and leading to death [1]. Currently, no effective treatment exists for these illnesses.

Every year, MNDs affect approximately 2 new cases per 100,000 people, and the prevalence of these disorders is about 5 to 7 cases per 100,000 [2]. MNDs are usually more common in men than women, and the incidence rate is 2:1 [3]. The life expectancy of patients with MND is quite variable: for about half of these disorders, death occurs 3 to 5 years from the onset of symptoms, but some people may live for more than 10 years, whereas in other cases the disease can be very rapidly progressive. Reasons accounting for such variability remain poorly understood.

MNDs can be classified in relation to the subpopulation of MNs affected mainly by the disease process as spinal muscular atrophy (SMA), progressive muscular atrophy, spinobulbar muscular atrophy (or Kennedy’s disease), and hereditary motor neuropathies involving lower MNs. Among them, SMA is the most common disease during childhood [4]. SMA is an autosomal recessive disease: the majority of patients with SMA carry mutations in the *SMN1* gene (survival motor neuron 1), resulting in the selective degeneration of lower α-MNs. The *SMN2* gene, an *SMN1* homologue, compensates for the abnormal production of SMN1 protein, and its levels of expression correlate with disease severity [5]. The pathology involves spinal cord MNs causing their degeneration and ultimately death.

Upper MNs are more vulnerable in primary lateral sclerosis, hereditary spastic paraplegias, and spinal muscular atrophy with respiratory distress type 1 [6].

Finally, disease processes that affect both upper and lower MN populations, such as amyotrophic lateral sclerosis (ALS), can be reported. ALS is mostly sporadic (SALS), and familial forms of ALS (FALS) account for 10% of cases [7]. *C9orf72* expansion has been observed in the majority of FALS cases [8], but many other genes have been involved in ALS etiopathogenesis such as Tar-DNA-binding protein 43 (*TDP-43*), fused in sarcoma protein (*FUS*) [9], and superoxide dismutase (*SOD1*) [10]. ALS incidence is 1 to 2 per 100,000 persons every year, and the age of onset is around 50 to 60 years: symptoms develop from paralysis to death within 2 to 5 years from the diagnosis [7].

Pathological mechanisms underlying the onset of ALS and generally MNDs are largely unknown. Many factors seem to be involved in the process with different contributions from environmental and genetic factors [11].

Stem cell-derived MNs represent a promising research tool in disease modeling, drug screening, and development of therapeutic approaches for MNDs and spinal cord injuries [12,13]. They could provide a replacement for dying cells and a trophic support within the central nervous system (CNS) [12].

In regard to the latter, stem cells may be supportive for endogenous cells modulating the diseased microenvironment by providing neurotrophic factors and scavenging toxic catabolites.

Particularly in the case of neurodegeneration, the potential positive effects due to cell replacement are related to the complexity of the pre-built host system. To effectively replace lost cells, transplanted ones should integrate within the host circuits and establish proper connections eventually reaching long-distance targets through the inhibitory white matter. So far, several transplantation strategies have focused on the more achievable ‘paracrine’ effect [14], in which transplanted cells act therapeutically through the secretion of diffusible factors. Several studies report a therapeutic effect of stem cells due to the trophic modulation of the neurodegenerated environment in different models of neurological disorders (that is, Parkinson’s disease, stroke, and Huntington’s disease) [15]. Proceeding beyond this strategy, stem cells can also be engineered to secrete selected molecules at the disease site [16]; human neural progenitor cells (hNPCs) modified by using lentivirus to secrete glial cell-derived neurotrophic factor (GDNF) integrated properly within ALS animal models. After transplantation into the spinal cord of *SOD1* (G93A) rats, a significant cell migration toward disease sites was observed together with efficient delivery of GDNF. A considerable preservation of MNs at early and end stages of the disease was shown within chimeric regions [17]. Similarly, hNPCs have been modified to release GDNF upon stimulation; cells have been transplanted in the striatum of a rodent model and could survive and effectively express GDNF, paving the way to further studies in Parkinson’s disease animal models [18].

Recent years have brought several advances in the stem cell field, concerning methods of both reprogramming and differentiation. Stem cells can be defined by their ability to replicate indefinitely while maintaining the capacity to form cells of the three germinal layers (ectoderm, endoderm, and mesoderm lineages). The several categories of stem cells reflect the wide range of cell types derivable and the ways in which stem cells are obtained.

Human embryonic stem cells (hESCs) are derived from the inner cell mass of the blastocyst, they are pluripotent, and they can be maintained *in vitro* for a good period of time with a stable genetic background, providing a source of specialized human cells for biological and clinical applications [19,20]. Directed differentiation of hESCs toward specific lineages is the first crucial step in order to extensively employ hESCs in early human development investigation as well as in potential future clinical applications. However, their use can raise ethical issues, and the therapeutic applications could imply risks of negative reaction, such as immune reactions or development of tumors or both [21].

Induced pluripotent stem cells (iPSCs) can be derived from patients’ somatic cells by reprogramming them with specific factors [22]. They can be considered embryonic stem cell (ESC)-like cells, which express stem cell markers and have the ability to give rise to all three germ layers, bypassing the ethical concerns. Specific individual human-derived iPSCs provide the premises for cell replacement therapy without the related risks of immune rejection. Thus, human induced pluripotent stem cells (hiPSCs) constitute an appealing alternative source for MN differentiation. Different methods have been reported for iPSC derivation. Viral methods (generally the most efficient ones) present major risks related to the stochastic activation/inactivation of endogenous genes. Non-integrative methods (that is, proteins, RNAs, mRNAs, and plasmids carrying the reprogramming factors) have been developed to bypass this concern and facilitate the transition to clinical practice [23].

During the last decades, increasing efforts have been made to direct specific differentiation of stem cells toward neuronal lineages. Data from several studies reported that ESCs and iPSCs are responsive *in vitro* to the same developmental stimuli guiding neural specification *in vivo* and are able to give rise to differentiated neuronal cells with specific morphological and molecular signatures. Obtaining fully differentiated cells is crucial to model in simplified *in vitro* platforms the complex processes underlying physiological development and disease pathogenetic mechanisms, with the ultimate aim to find a cure for orphan disorders.

This could be particularly important for those pathologies, such as MNDs, for which obtaining affected relevant cells from human patients can be challenging. Indeed, some of the first reported examples about generating human disease-specific cells are related to MNDs [24,25]. Many issues need to be addressed to effectively translate iPSCs to large-scale preclinical studies; among them is the assessment of standardized efficient protocols of differentiation and of homogenous parameters to evaluate the obtained cell phenotype. In the case of spinal MNs, efforts aiming to point out rapid and highly efficient differentiation methods are being pursued worldwide.

In the present review, we will first describe the fundamental steps regulating MN specification *in vivo*; data obtained from these studies have guided the assessment of experimental differentiation protocols *in vitro*. We will focus our attention on MN differentiation derived from hESCs and iPSCs; the most used methods of neural induction will be reviewed. Neural induction is the first crucial step to obtain neural progenitors through neural rosette and embryoid body (EB) formation. Then, neural progenitors can be differentiated with different protocols varying for rapidity and efficiency. We will give an overview of the most recent published methodologies together with the different methods of evaluation of obtained MNs. Finally, we will consider potential clinical applications of hiPSC/ESC-derived MNs with a focus on MND modeling and treatment, paying attention to the biological challenges that we need to address before translating promising preclinical data into clinical application.

## Motor neuron development

**Figure 1:**
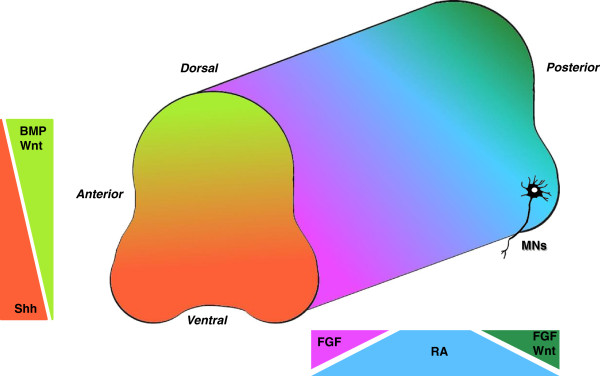
**Schematic representation of the role of morphogens during neural tube formation *in vivo*.** Color gradients are indicative of the expression levels of each morphogen. Bone morphogen protein (BMP) can be found in high concentrations in the dorsal part of the neural tube (light green): its levels decrease along the ventral part. In contrast, Sonic hedgehog (Shh) is more concentrated in the ventral part (orange), but it is not expressed in the dorsal one. Fibroblast growth factor (FGF) is highly expressed in the anterior (purple) and posterior (dark green) parts of the neural tube. Retinoic acid (RA) levels of expression decrease in the posterior part (light blue), where high concentrations of both FGF and Wnt can be found. MN, motor neuron.

During embryogenesis, patterning and cell fate specification are regulated by the local production and secretion of protein morphogens and growth factors. Neurogenesis is the developmental process by which the whole nervous system is generated; it starts from the ectodermal plaque that folds over on itself, giving rise to the neural tube. The neural tube grows along rostrocaudal and dorsoventral axes, and during this process the cellular differentiation is induced by morphogenetic factors and signaling molecules secreted by neighboring cells (Figure 1) [26]. Morphogens induce the activation of intracellular pathways that, through the activation of specific transcriptional factors, cause a specific genetic expression that leads to cellular differentiation. In particular, specification of spinal MN fate is determined by three steps: neuralization, caudalization, and ventralization [27].

The term neuralization refers to the specification of the ectodermal cells toward epidermis or neural fate. This specification is influenced by the bone morphogen protein (BMP) that belongs to the transforming growth factor-beta (TGF-β) superfamily of proteins [28]. Therefore, neural induction is initiated through inhibition of both BMP and TGF-β/SMAD signaling [29]. Spinal MNs are diversified along the rostrocaudal axis by retinoic acid (RA) [30], which induces neuralization and caudalization and Wnt and fibroblast growth factor (FGF) signals [31] that promote an anterior or rostral pattern [32]. Ventral patterning is controlled by Sonic hedgehog (Shh) that directs ventralization of the spinal neural progenitor cell [33], whereas dorsal patterning is controlled by members of the BMP family [34].

BMP factors are selectively located in the ‘roof plate’ regions of the dorsal neural tube. Other growth factors are expressed specifically by the most ventral region (‘floor plate’). The roof plate gives rise to the interneurons, whereas the region of floor plate is the site of origin of developing MNs. The dorsoventral axis of the neural tube is established by Wnt morphogenetic signaling gradients: high concentration of Wnt determinates the dorsal region, whereas Shh signaling defines the ventral region [35]. Wnt proteins are also involved in axon guidance within the spinal cord in an anterior-posterior direction [36].

Understanding pathways and specific morphogens that regulate MN development is crucial to replicate *in vitro* some of these physiological steps in order to obtain enriched MN populations starting from human iPSCs and ESCs. At present, there are many protocols that exploit morphogens or synthetic agonists to promote MN specification [37,38].

## Motor neuron generation from human pluripotent stem cells

Different methods for MN differentiation have been performed (Table 1); here, we summarize a selection of the recently published and most significant protocols for obtaining a highly pure population of MNs differentiated from human pluripotent stem cells (hESCs and hiPSCs).

**Table 1:** Experimental protocols for motor neuron induction from human embryonic and induced pluripotent stem cells

**References**	**Starting cells**	**Neural induction**			**MN differentiation**		**MN maturation**		**Duration**
		**Medium**	**Factors**	**Obtained cells**	**Medium**	**Factors**	**Medium**	**Factors**	
Amoroso *et al*. [39]	ESCs and iPSCs	ESC medium^a^	Y27632, bFGF, LDN193189, and SB435142	Embryoid bodies	Neural induction medium^b^	RA, ascorbic acid, BDNF, C25II modified Shh, SAG, HAG, or purmorphamine	Neurobasal medium	IGF1, GDNF, CNTF, and B27	20-30 days
Boulting *et al*. [40]	ESCs and iPSCs	ESC medium with 10% KO replacement	bFGF and Y27632	Embryoid bodies	Neural induction medium^b^ supplemented with bFGF	RA, ascorbic acid, db-cAMP, and HAG	Neurobasal medium	BDNF, GDNF, and CNTF	29 days
Burkhardt *et al*. [41]	iPSCs	DMEM/F12 supplemented with N2, B27, and NEAA	Y27632, Dorsomorphin dihydrochloride	Embryoid bodies	DMEM/F12, GLUTAMAX, N2, B27 serum-free supplement, D-Glucose, and ascorbic acid	SAG, RA, and DAPT (only for 4 days)	DMEM/F12, GLUTAMAX	CNTF, BDNF, and GDNF	32 days
							N2, B27 serum free supplement, D-Glucose, and ascorbic acid		
Corti *et al*. [25]	iPSCs	DMEM/F12, NEAA, N2, and heparin	RA	Neural rosette	DMEM/F12, NEAA, N2, and heparin	RA and Shh	DMEM/F12, NEAA, N2, and heparin	BDNF, GDNF, and IGF1	~24 days
Hester *et al*. [42]	ESCs and iPSCs	DMEM/F12, N2, and 10% KO serum	-	Embryoid bodies then neural rosette	DMEM/F12 with N2	RA, Shh, forskolin, B27, and adenovirus	-	-	~40 days
Hu and Zhang [43]	ESCs	DMEM/F12, KO serum replacement, NEAA, L-Glu, and BME	-	Floating embryoid bodies	Neural differentiation medium (DMEM/F12, N2, NEAA, and heparin)	Shh or purmorphamine, RA, B27, cAMP, ascorbic acid, BDNF, GDNF, and IGF1	Neural differentiation medium (DMEM/F12, N2, NEAA, and heparin)	cAMP, ascorbic acid, BDNF, GDNF, and IGF1	~40 days
Karumbayaram *et al*. [44]	iPSCs and ESCs	ESC medium without FGF2	-	Embryoid bodies/neural rosette	ESC medium without FGF2	RA and purmorphamine	DMEM/F12 and N2	GDNF, BDNF, CNTF, Shh, and RA	35-49 days
Reinhardt *et al*. [29]	iPSCs and ESCs	ESC medium^a^ and then expansion medium (DMEM/F12, N2, B27, pen/strep, and L-Glu)	For induction: SB435142, Dorsomorphine, CHIR, and purmorphamine	Embryoid bodies (smNPC)	Expansion medium (DMEM/F12, N2, B27, pen/strep, and L-Glu)	Purmorphamine and RA	Expansion N2, B27	BDNF, GDNF, db-cAMP, RA, and purmorphamine	>40 days
			For expansion: ascorbic acid, CHIR99021, and purmorphamine						
Sareen *et al*. [45]	iPSCs	Neural differentiation medium (DMEM, B27, vitamin A, and N2)	RA	Embryoid bodies	Neurobasal medium, B27, and N2	RA and purmorphamine	DMEM/F12 and B27	RA, purmorphamine, db-cAMP, ascorbic acid, BDNF, and GDNF	>40 days
Takazawa *et al*. [37]	ESCs	ESC medium^a^ and then DMEM/F12, N2, NEAA, L-Glu, and heparin	Y27632, bFGF, and recombinant mouse Noggin	Embryoid bodies	Wnt3a-L-cell conditioned medium	RA, ascorbic acid, db-cAMP, and recombinant mouse Shh (C25II)	Neurobasal medium with N2, B27, Glu, and NEAA	Ascorbic acid, db-cAMP, RA, Shh, BDNF, GDNF, and IGF1	>31 days
Wada *et al*. [46]	ESCs	ESC medium^a^ and FGF2	Noggin and dorsomorphin	Neural rosette	DMEM/F12, N2B27	Shh or SAG, RA	DMEM/F12, N2, B27, FGF2, and heparin	GDNF, BDNF, and NT3	38 days
					FGF2, and EGF				
Wichterle *et al*. [27]	mESCs	DFK5 medium (DMEM/F12, L-Glu, pen/strep, BME, and insulin-transferrin- selenium supplement	RA, Shh, Hedgehog agonist (Hh-Ag1.3), or hedgehog antibody (5E1)	Embryoid bodies	DMEM/F12 medium	-	DMEM/F12	GDNF, BDNF, CNTF, and NT3	~25 days
Zeng *et al*. [47]	iPSC (ESCs as positive control)	hESC medium^a^	N2 and heparin	Neural rosette	Neural medium^b^	RA, Shh, and FGF8	Neural basal medium, N2, and B27	GDNF, BDNF, and IGF1	>24 days

^a^Human embryonic stem cell (hESC) medium: Dulbecco’s modified Eagle’s medium (DMEM/F12), 20% knockout (KO) serum replacement, basal medium Eagle (BME), L-Glu, and non-essential amino acid (NEAA). ^b^Neural induction medium: L-Glu, NEAA, penicillin/streptomycin (pen/strep), heparin, and N2. BDNF, brain-derived neurotrophic factor; bFGF, basic fibroblast growth factor; CNTF, ciliary neurotrophic factor; EGF, epidermal growth factor; ESC, embryonic stem cell; FGF, fibroblast growth factor; GDNF, glial cell-derived neurotrophic factor; HAG, human-specific Smo agonist; IGF1, insulin-like growth factor-1; iPSC, induced pluripotent stem cell; mESC, murine embryonic stem cell; MN, motor neuron; RA, retinoic acid; SAG, Smo agonist; Shh, Sonic hedgehog; smNPC, small-molecule neural precursor cell.

### Neural induction

**Figure 2:**
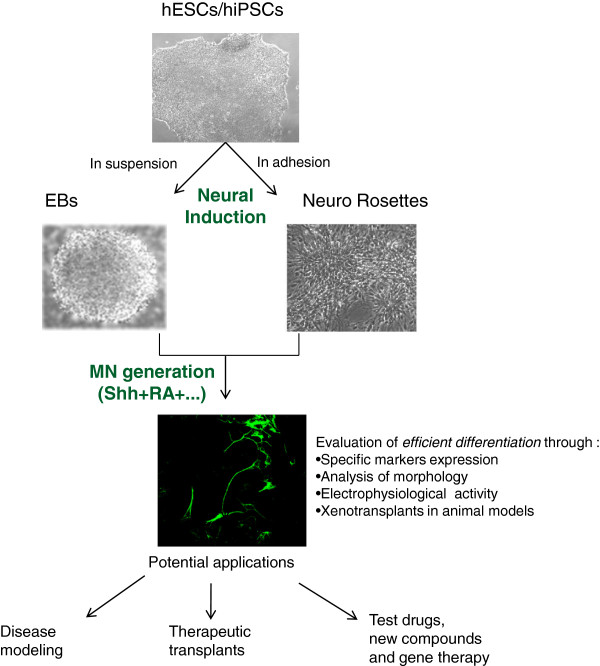
**Generation of human motor neurons from human embryonic and induced pluripotent stem cells.** A schematic representation for motor neuron (MN) generation *in vitro* is shown. The first step in pluripotent stem cell - human embryonic stem cell (hESC) and human induced pluripotent stem cell (hiPSC) - differentiation is the attainment of embryoid bodies (EBs) in suspension or neural rosettes in adhesion conditions. These neural precursors can be successfully differentiated in MNs (characterized by specific features) with different multistage experimental protocols. hESC- or iPSC-derived MNs are a promising research tool to model and study *in vitro* pathological mechanisms underlying MN diseases in humans. These MNs could also represent an appealing source for autologous cell replacement. RA, retinoic acid; Shh, Sonic hedgehog.

The term neural induction refers to the formation of neural progenitors deriving from pluripotent stem cells. There are several methods to promote neural induction; the majority include neural rosette and EB formation (Figure 2).

Neural rosettes can be considered a stage at which neural stem cells exhibit a high proliferative potential and broad differentiation capacity along both neuronal and glial lineages in response to appropriate developmental signals [48]. Neural rosettes are defined by their specific cytoarchitecture, gene expression, and extrinsic growth requirements. To generate neural rosettes, hESCs and iPSCs are usually seeded on poly-L-lysine/laminin-coated culture dishes and maintained under adhesion conditions. Cells are usually plated in neurobasal medium supplemented with N2 and B27 for the growth and long-term viability of post-mitotic neurons. The medium can be additionally supplemented with mouse/human recombinant Noggin [37,46] or Dorsomorphin, which are employed as inhibitors of BMP signaling to enhance neural induction [29,38,46]. Alternatively, stem cells can be cultured on stromal feeder [49] or transferred in modified TeSR1 medium until cells adhere and rosette structures appear [50].

Reinhardt and colleagues [29] reported the generation of hNPCs using only small molecules. These neural progenitors do not require manual selection and they are also able to differentiate into neural tube lineages, including MNs. The authors also hypothesized that Wnt, in combination with Shh, might contribute to the maintenance of neural precursors. Neural induction was promoted through inhibition of both BMP and TGF-β signaling using Dorsomorphin and SB43152. To stimulate the canonical Wnt signaling, CHIR99021, a GSK3β inhibitor, was added to the cell medium and the Shh pathway was stimulated by using purmorphamine.

In recent years, many groups have exploited protocols focused on the formation of EBs to differentiate stem cells into MNs. EBs are three-dimensional cell aggregates that grow spontaneously, self-assembling in suspension cultures. For EB formation, iPSCs or ESCs are detached from mouse embryonic feeder fibroblasts and cultivated onto ultralow adherent culture dishes. Cell culture medium is similar to the one used for hESC culture, supplemented with TGF-β and BMP inhibitors. Media usually contain Noggin or a small-molecule substitute, like Y-27632 [51] to increase cell survival, SB435142 and LDN193189 to promote neural induction [39], and FGF to enhance growth.

Some authors adopted a combination of the two methods: EBs were cultivated in a suspension environment and then the aggregates were dissociated and seeded on laminin/poliornithin-coated plates to form neural rosettes [25,43].

### Motor neuron generation

Thanks to the cultural background of developmental biology, it is known that MN differentiation *in vitro* appears to summarize the sequence of events physiologically involved in spinal cord development. In 2002, Wichterle and colleagues [27] were the first ones to exploit RA and Shh to differentiate mouse ESCs through EB formation; MN differentiation protocols for rodent ESCs were later adapted to promote MN commitment of hESCs as well. Wada and colleagues [46] differentiated hESCs toward MNs through neural rosette formation: neural precursors derived from hESCs were treated with 1 μM RA and 500 ng/mL Shh, resulting in large numbers of Tubulin β III^+^, Hb9^+^, Islet1^+^, and choline acetyltransferase-positive (ChAT^+^) neurons. Transcriptional upregulation of MN markers such as Islet 1, Hb9, and Olig2 was shown. Terminally differentiated neurons were Synapsin-positive and electrophysiologically active. MNs were capable of recreating neuromuscular junctions in culture with C2C12 myotubule cells. Overall, these data indicate that hESCs can differentiate into MNs that express specific molecular markers and have functional properties similar to those of physiologically developed MNs. Through the years, many reported protocols have tested different efficacious concentrations of the crucial signaling molecules, from 1 nM to 1 μM of RA and from 50 to 500 ng/mL of Shh [25,39,40,45].

Another crucial variable to be modulated is the time of addition of the small molecules for differentiation. Hu and Zhang [43] reported a protocol starting from hESCs that were differentiated into MNs: cells were cultured for 4 days in hESC medium and in neural differentiation medium thereafter. In the second week, aggregates attached to the surface of culture dishes and possessing features of neuroepithelial rosettes could be isolated by manual selection. The authors reported that the early neuroepithelial cells Olig2-positive at day 10 were much more responsive to RA; thus, RA was added at day 10 in their protocol. These Olig2-expressing cells then differentiated to spinal motoneurons in the fifth week and expressed transcription factors such as Hb9 and Islet1. They reported that the optimized protocol typically generates over 50% of Hb9-expressing motoneurons from the original hESC progenies.

Another variable to be considered is the natural habit of human stem cells to differentiate into caudal/rostral subtypes giving rise to medial/lateral column as well as cranial MNs. Similarly, the selective differentiation signal inducing the development of specific neuronal subtypes within the wide range of spinal cord MNs warrants further studies to be extensively applied in *in vitro* studies and cell therapies. The manipulation of Hox gene network specifying columnar and pool MN identity may be fundamental to selectively modulate the differentiation of precursor cells into specific MN subtypes. Recently, Amoroso and colleagues [39] systematically compared the ventralizing activity of three Smoothened agonists using a standard RA/Shh protocol involving all-trans RA and a modified Sonic hedgehog (Shh-C25II) protein as a benchmark for differentiation of hESCs into MNs. To evaluate MN numbers, they relied on the HUES3 Hb9:GFP reporter line which contains a transgene expressing green fluorescent protein (GFP) under the control of the MN-specific Hb9 promoter. HUES3 cells were electroporated with a plasmid carrying a neomycin resistance cassette and the GFP coding sequence upon transcriptional control of Hb9 promoter restriction fragment. In this way, stem cells were engineered in order to express GFP under the control of the MN-specific reporter Hb9. This strategy allows the study of cell morphology and differentiation in culture and their tracing *in vivo*. However, it has to be considered that this marker does not provide any information on motor neuronal subtype and the fluorescence of GFP could give false-positive results due to the GFP long half-life. Very few GFP^+^ cells were observed in the absence of exogenous Shh agonists. Recombinant Shh and human-specific Smo agonist each gave rise to less than 10% GFP^+^ cells. In contrast, the Smo agonist (SAG) alone gave rise to 16% ± 4% GFP^+^ cells and purmorphamine alone induced 22% ± 6%. RNA-seq analyses revealed an enrichment of spinal MN markers and the expression of cholinergic genes in cells treated with SAG and purmorphamine compared with only Shh-treated ones. This work exploited non-viral protocols of differentiation, characterized also by relatively high rapidity and efficiency. Not less important, Amoroso and colleagues [39] made great efforts in selecting and validating a pool of specific markers to evaluate the cell phenotype: Hb9 and ISL1 were found to be alternatively expressed in approximately half of the cells, not always co-expressed as originally believed. Moreover, this differentiation method starting from hESCs resulted in the generation of MNs expressing FOXP1 (68% ± 4%), which is a marker of limb muscle-innervating lateral motor column neurons. This regional sub-specification could be important for disease modeling studies.

Many other differentiation cocktails have been reported to share the objective of gaining highly enriched spinal cord motor neuronal populations: supplementing RA and Shh with B27 [43,46], cAMP [37,40,45], heparin, brain-derived neurotrophic factor (BDNF), and ascorbic acid [37,40,45]. López-González and colleagues [52] analyzed the effect of progesterone and 17β-estradiol on MN differentiation of HBG3 ESCs. Progesterone treatment during MN differentiation at EB stage, combined with RA and Shh, induced higher proportions of MN compared with RA/Shh alone [52].

The discovery and establishment of the use of iPSCs provided further advances in the field; it has become possible, by using hiPSCs, to produce MNs carrying the specific combination of genetic variants that caused neurodegeneration in a single patient. A multistage MN differentiation protocol starting from SMA patient-derived iPSCs has been performed by Corti and colleagues [25]. For MN generation, iPSCs derived from patients with SMA were grown in a neuronal medium supplemented with N2 and heparin. After 10 days, RA (0.1 μM) was added for caudalization, and at day 17 the clusters of posteriorized neuroectodermal cells were resuspended for a week in the same medium with RA (0.1 μM) and Shh (100 to 200 ng/mL). On day 24, BDNF, GDNF, and insulin-like growth factor-1 were added for MN maturation. Derived human MNs were characterized by the expression of specific markers and carried the pathological hallmarks of SMA disease.

This study provided important data on the potential applications of stem cells both as *in vitro* models of disease and as a feasible therapeutic approach. In regard to the former, generated MNs presented features indicative of mature lineage and spinal MN commitment such as HB9/ISLET1 and SMI32. The *in vitro* differentiation protocol generated a mixed cell population, including non-motor neuronal cells. To further isolate and purify MNs, a physical strategy based on gradient centrifugation and leading to an enriched MN population was exploited [25]. Moreover, SMA-iPSC-derived MNs showed shorter axonal length, smaller growth cones, and impaired ability to form neuromuscular junctions compared with wild-type cells, hallmarks of MN disease. In regard to the latter, reprogrammed cells have been obtained with non-viral non-integrating methods, which could be suitable for human therapy uses in the future. Once reprogrammed cells were transplanted into the mouse model, there were no major signs of rejection but rather a proper engraftment was documented with even a partial rescue of the diseased phenotype.

Similarly, different methods have been performed for MN induction starting from hiPSCs derived from patients affected by ALS. Sareen and colleagues [45] generated iPSC-derived MNs from patients with ALS by growing iPSCs for 6 days in suspension with neural differentiation medium enriched with RA for EB formation. At day 17, cells were treated with neural induction medium supplemented with RA and purmorphamine for 8 days. Then EBs were dissociated and single cells were plated in medium with B27, RA, purmorphamine, db-cAMP, ascorbic acid, BDNF, and GDNF for 2 to 7 weeks. Derived MNs recapitulate the features of ALS disease and can be employed to investigate cellular degeneration processes and test new therapeutic compounds.

### Derived motor neuron evaluation

The increasing expertise in differentiating MNs with different experimental protocol variables emphasizes the necessity for continuous evaluation of derived MNs. To exploit human stem cell-derived MNs in regenerative medicine or in MND *in vitro* modeling, it is first necessary to evaluate their proper differentiation, assessing that they have the same features as MNs *in vivo*. An efficient MN differentiation starting from human ESC and iPSC lines can be assessed by evaluation of those features that are the hallmarks of MNs *in vivo* (that is, marker expression, morphology, and functional properties) (Figure 2). The authors demonstrated that hESC- and hiPSC-derived MNs are characterized by the expression of the same specific markers previously assessed in human MNs *in vivo*[25-27]. Indeed, the expression of MN post-mitotic markers such as Hb9, HoxC8, ChAT, and SMI-32 was evaluated by immunocytochemistry assays, as was the expression of MN progenitor markers like Pax6, Nestin, Olig2, and Islet1/2 and pan-neural markers such as β-tubulin and anti-microtubule-associated protein 2 [25,29,39,42,45].

Karumbayaram and colleagues [44] demonstrated that MNs derived through neural rosette and EB formation expressed MN progenitor markers (that is, Olig2 and Nkx6.1) and also showed an enrichment of mature markers like β-tubulin, ChAT, and Islet1. To further characterize the expression of these markers in an unbiased manner, authors usually perform Western blot, PCR - quantitative reverse transcription-PCR and reverse transcription-PCR - and whole-transcriptome sequencing or RNA-seq to detect an enrichment of spinal MN markers (Islet1, Islet2, and Hb9) and of CHAT, CHT1, VACHT, CHRNA3, CHRNA4, and CHRNB2, which are indicative of cholinergic identity [27,39].

In several studies, stem cells were manipulated in order to express GFP driven by an MN-specific reporter. A reporter specific for activity of Hb9 which encodes for a transcription factor specifically expressed by mature MNs is most commonly used because of its relatively high specificity and assessed staining.

Hb9-driven GFP reporter is usually transfected into stem cells, allowing the identification of MNs in which Hb9 is transcriptionally active. This technique facilitates cell study in culture and cell tracing *in vivo*. Hb9:GFP stem cells can be studied for their maturation in cellular morphology (that is, branching and neurite outgrowth) and increased soma area over time [29,39,44]. Other authors developed Olig2:GFP lines in which an enhanced GFP cassette was inserted to the Olig2 locus of hESCs to target MN progenitors [53]. Electrophysiological analysis, including calcium imaging and whole-cell patch clamp, showed that stem cell-derived MNs become electrically active with prolonged time in culture and are responsive to glutamate agonist [27,34,38,39,49]. Takazawa and colleagues [37] observed electrophysiological changes associated with maturation of MNs differentiated from stem cells, which included decreasing input resistance and increasing action potential firing frequency. Furthermore, these cells showed two peculiar characteristics of spinal MNs *in vivo*: spike frequency adaptation and rebound action potential firing [37].

*In vivo* morphological analyses can consist of xenotransplants by injection of stem cell-derived MNs (usually Hb9:GFP^+^ to be easily traced) into animal models. Pluripotent stem cell-derived MNs demonstrated the ability to project axons outside of the CNS through the ventral (and sometimes dorsal) roots and to follow proper neural paths when transplanted into spinal cord of developing chickens [39] or mice [25,29]. Furthermore, Corti and colleagues [25] demonstrated that engrafted GFP-MNs co-expressed pan-neuronal-specific markers and ChAT, formed new neuromuscular junctions with skeletal muscles, and, once transplanted into SMA model mice, induced an improvement of neuromuscular phenotype and survival.

## Clinical perspectives

### Disease modeling

Disease modeling represents an interesting possibility of application of human stem cell-derived MNs: with the optimization of standardized protocols for stem cell differentiation, it has become achievable to produce human MNs in substantial amounts for elucidating *in vitro* basic disease processes. Stem cells could also be manipulated in order to express mutant genes related to MNDs [54,55].

The discovery of reprogramming methods using specific factors and the subsequent derivation of the first patient-specific hiPSC lines provided a further step in this direction.

iPSCs can be employed to derive human MNs *in vitro* and thus address specific questions about altered neuronal differentiation and function that might cause the development of diseases such as ALS, SMA, and many others. The possibility to recreate *in vitro* a reliable model of human disease is valuable, as there have been many examples of therapeutic approaches that were relatively efficacious in animal models but unfortunately did not translate well to patients [7].

Burkhardt and colleagues [41] reprogrammed fibroblasts to iPSCs derived from a large cohort of healthy controls and ALS patients and differentiated them into MNs. The authors reported that MNs derived from three patients with SALS possessed *de novo TDP-43* aggregation and that these aggregates summarized pathological features in post-mortem tissue from one of the three patients from which iPSCs were derived. The authors then performed a high-content chemical screen by using the TDP-43 aggregate endpoint in both lower MNs and upper derived MNs identifying US Food and Drug Administration-approved small-molecule modulators.

Corti and colleagues [25] generated iPSCs from SMA patients with non-viral methods resulting in cells free from vectors and transgenic sequences. SMA-iPSCs were then differentiated into MNs with a multistage differentiation protocol involving RA and Shh. MNs generated from SMA-iPSCs carried specific disease-related features suggestive of selective MN degeneration such as a reduction in MN survival and size as well as in axonal growth and neuromuscular junction formation. Oligodeoxynucleotides to SMN2 were generated with a stable genetic modification of a single nucleotide in exon 7, leading to the modification of SMN2 coding region. As a consequence, exon 7 was rescued, resulting in a greater production of full-length SMN2. Phenotypes of MNs derived from corrected and untreated SMA-iPSCs were compared: gene correction with oligonucleotides rescued neuropathological features in SMA MNs and correlated with SMN expression. Transcriptional differences in the SMA-iPSC MNs in comparison with treated SMA-iPSC MNs were evaluated, revealing alterations in a subset of genes involved in RNA metabolism, MN development, and axonal guidance.

Thus far, various iPSC lines from patients with various neurodegenerative disorders, including Alzheimer’s disease, Parkinson’s disease, and Huntington’s disease, have been generated [56-58]. Patient-derived stem cells could be one of the best complementary approaches to the use of reliable animal models to identify and test therapeutic compounds for neurological disorders.

### Therapeutic transplants of human motor neurons

Cell therapy in neurodegenerative disorders acts by introducing functional cells in order to rescue the function of damaged neural tissues. Transplantation of hESC-derived MNs into the developing chick embryo resulted in correct engraftment, maintenance of motoneuron identity and long-distance axon elongation outside the CNS, reaching properly peripheral muscular targets [49]. Transplantation into the adult rat spinal cord resulted in neural engraftment, including a great number of human MNs with sprout of ChAT^+^ fibers. These data suggest that hESC-derived MNs may be able to project toward the ventral root through the adult spinal cord, similarly to the embryonic chick spinal cord even if with a different time scale. This study provided evidence for *in vivo* survival of hESC-derived MNs, a crucial requirement for future preclinical applications.

hiPSCs are similar to hESCs, and the experimental strategies developed for hESCs could be applied to iPSCs without major modifications. In addition, hiPSCs do not have issues in the immunologic compatibility between donors and recipients reported in hESCs [57]. Thus, they have been investigated as a promising source of autologous cells for transplant therapy in neurological disorders. iPSCs from patients with SMA (SMA-iPSCs) were generated by using non-viral, non-integrating episomal vectors: *SMN2* gene was converted into an *SMN1*-like gene with a strategy based on the use of single-stranded oligonucleotides [25]. *In vivo* experiments after transplantation into the spinal cord of transgenic SMA mice showed that iPSC-derived MNs can survive and integrate into the spinal cord of SMA mice and ameliorate the SMA type I phenotype. Transplanted SMA mice had a longer survival (about 50%) compared with vehicle-treated mice, a beneficial effect that was more relevant with treated SMA-iPSC MNs than with untreated ones [25].

These appealing premises for clinical therapies have to be modulated by the presence of crucial issues to be addressed before effectively translating cell-mediated approaches to the clinic. Important concerns are related to the generation and preparation of cells under good manufacturing practice (GMP). It has been demonstrated that ESCs can be generated without the use of reagents of animal origin (xenobiotics); however, the derivation of patient-specific iPSCs usually requires methods of reprogramming that are not performed in xeno-free conditions. iPSCs generated with viral methods present major risks related to the stochastic activation/inactivation of endogenous genes. Non-integrative methods (that is, proteins, RNAs, mRNAs, and plasmids carrying the reprogramming factors) have been improved to bypass this concern and facilitate the transition to the clinic [23]. Neural stem cells to be transplanted can be obtained also by direct reprogramming (induced neural stem cells, or iNSCs): experimental protocols present fewer passages in comparison with the generation of iPSCs, thus lessening the necessity of different quality-check points [59]. On the other hand, iNSCs are a rather new source of stem cells and consequently generation methods still need to be standardized together with a precise evaluation of the obtained phenotype [59]. In general, protocols of differentiation toward a specific lineage, including rigorous methods of data homogenization and standardization in order to be reproducible, should be implemented. Several xeno-free media preparations are commercially available, together with GMP feeders [23]. Differentiated phenotype needs to be rigorously evaluated, and different assessment methods are usually combined to limit eventual unspecific staining. Indeed, GMP optimized techniques to trace and evaluate the state of transplanted cells will be necessary after clinical translation. Finally, obtained cells should undergo standardized quality control to assess viability, sterility, and proper cell conformation (that is, karyotype analysis, specific marker evaluation, and absence of xenobiotic contamination) [23].

Concerns related to the host immunorejection could be bypassed with the use of autologous cells, such as iPSCs. However, studies rigorously assessing iPSC immunogenicity still need to be performed, and results could vary on the basis of cell preparation protocols.

The clinical perspectives on the use of cell-based therapies have to be calibrated on the basis of the previous issues: the premises for future treatments are great but need to be thoroughly tested by standardized *in vitro* studies at first and then by a rigorously assessed clinical trial in humans.

Despite many aspects to be cleared and certainly worthy of further studies (that is, ability of transplanted MNs to survive, integrate, and project their axons for long-distance innervating properly peripheral targets), these preliminary results open the way to a future in which human pluripotent stem cells may provide a source of healthy MNs for therapeutic transplantation for SMA and other MNDs.

## Conclusions

The potential of human pluripotent stem cells to treat patients with neurodegenerative disease is enormous. Stem cells can be introduced into clinical applications in several different ways, such as disease modeling, drug screening, and cell replacement therapy.

The development and optimization of non-viral methods of stem cell reprogramming will allow clinicians to bypass concerns related to random viral integration. The use of autologous patient cells could also alleviate problems associated with host immunorejection. In regard to the production of differentiated cells, it is crucial to assess protocols with high efficiency allowing a large-scale production for quality-control assessment. A very recently published study reported the generation of MNs from iPSCs in 20 days [60]. In view of future clinical applications, it is necessary to integrate efficiency and rapidity of production with constant attention to working under GMP conditions, exploiting xeno-free media, and conducting a careful quality-control assessment. Recent progress in stem cell research has opened new perspectives for *in vitro* generation of large numbers of various neural cell types and for their use in the repair of the nervous system. Advances in obtaining and understanding MNs have been made in recent years; however, some aspects still need to be investigated. Among them, the molecular signature of MN development and specification into different subtypes *in vivo* is only partially known. In regard to different methods exploited to produce MNs, extensive applications need a precise consideration of the experimental conditions for cell plating and culturing. Moreover, challenges such as those ensuring clinical safety should be overcome. Even though it may take quite a long time to address all of these questions, the establishment and optimization of human stem cell differentiation protocols to develop hiPSC-based clinical applications may hold the key to curing neurodegenerative disorders.

## Abbreviations

ALS: Amyotrophic lateral sclerosis; BDNF: Brain-derived neurotrophic factor; BMP: Bone morphogen protein; ChAT: Choline acetyltransferase; CNS: Central nervous system; EB: Embryoid body; ESC: Embryonic stem cell; FALS: Familial amyotrophic lateral sclerosis; FGF: Fibroblast growth factor; GDNF: Glial cell-derived neurotrophic factor; GFP: Green fluorescent protein; GMP: Good manufacturing practice; hESC: Human embryonic stem cell; hiPSC: Human induced pluripotent stem cell; hNPC: Human neural progenitor cell; iNSC: Induced neural stem cell; iPSC: Induced pluripotent stem cell; MN: Motor neuron; MND: Motor neuron disease; PCR: Polymerase chain reaction; RA: Retinoic acid; SAG: Smo agonist; SALS: Sporadic amyotrophic lateral sclerosis; Shh: Sonic hedgehog; SMA: Spinal muscular atrophy; SMN: Survival motor neuron; *SOD1*: Superoxide dismutase; *TDP-43*: Tar-DNA-binding protein 43; TGF-β: Transforming growth factor-beta.

## Competing interests

The authors declare that they have no competing interests.

## Authors’ contributions

All authors read and approved the final manuscript.

## References

[B1] Mitne-Neto M Machado-Costa M Marchetto MC Bengtson MH Joazeiro CA Tsuda H Bellen HJ Silva HC Oliveira AS Lazar M Muotri AR Zatz M Downregulation of VAPB expression in motor neurons derived from induced pluripotent stem cells of ALS8 patients Hum Mol Genet 2011 20 3642 3652 10.1093/hmg/ddr284 21685205 PMC3159551

[B2] McDermott CJ Shaw PJ Diagnosis and management of motor neurone disease BMJ 2008 336 658 662 10.1136/bmj.39493.511759.BE 18356234 PMC2270983

[B3] Orrell RW GPs have key role in managing motor neurone disease Practitioner 2011 255 19 22 22032111

[B4] D’Amico A Mercuri E Tiziano FD Bertini E Spinal muscular atrophy Orphanet J Rare Dis 2011 6 71 10.1186/1750-1172-6-71 22047105 PMC3231874

[B5] Lefebvre S Burglen L Reboullet S Clermont O Burlet P Viollet L Benichou B Cruaud C Millasseau P Zeviani M Le Paslier D Frézal J Cohen D Weissenbach J Munnich A Melki J Identification and characterization of a spinal muscular atrophy-determining gene Cell 1995 80 155 165 10.1016/0092-8674(95)90460-3 7813012

[B6] Sau D Rusmini P Crippa V Onesto E Bolzoni E Ratti A Poletti A Dysregulation of axonal transport and motorneuron diseases Biol Cell 2011 103 87 107 10.1042/BC20100093 21250942

[B7] Gordon PH Amyotrophic lateral sclerosis: an update for 2013 clinical features, pathophysiology, management and therapeutic trials Aging Dis 2013 4 295 310 10.14336/AD.2013.0400295 24124634 PMC3794725

[B8] DeJesus-Hernandez M Mackenzie IR Boeve BF Boxer AL Baker M Rutherford NJ Nicholson AM Finch NA Flynn H Adamson J Kouri N Wojtas A Sengdy P Hsiung GY Karydas A Seeley WW Josephs KA Coppola G Geschwind DH Wszolek ZK Feldman H Knopman DS Petersen RC Miller BL Dickson DW Boylan KB Graff-Radford NR Rademakers R Expanded GGGGCC hexanucleotide repeat in noncoding region of C9ORF72 causes chromosome 9p-linked FTD and ALS Neuron 2011 72 245 256 10.1016/j.neuron.2011.09.011 21944778 PMC3202986

[B9] Lattante S Rouleau GA Kabashi E TARDBP and FUS mutations associated with amyotrophic lateral sclerosis: summary and update Hum Mutat 2013 34 812 826 10.1002/humu.22319 23559573

[B10] Rotunno MS Bosco DA An emerging role for misfolded wild-type SOD1 in sporadic ALS pathogenesis Front Cell Neurosci 2013 7 253 24379756 10.3389/fncel.2013.00253 PMC3863749

[B11] Limpert AS Mattmann ME Cosford ND Recent progress in the discovery of small molecules for the treatment of amyotrophic lateral sclerosis (ALS) Beilstein J Org Chem 2013 9 717 732 23766784 10.3762/bjoc.9.82 PMC3678841

[B12] Lunn JS Sakowski SA Federici T Glass JD Boulis NM Feldman EL Stem cell technology for the study and treatment of motor neuron diseases Regen Med 2011 6 201 213 10.2217/rme.11.6 21391854 PMC3154698

[B13] Amemori T Romanyuk N Jendelova P Herynek V Turnovcova K Prochazka P Kapcalova M Cocks G Price J Sykova E Human conditionally immortalized neural stem cells improve locomotor function after spinal cord injury in the rat Stem Cell Res Ther 2013 4 68 10.1186/scrt219 23759119 PMC3706805

[B14] Rossi F Cattaneo E Opinion: neural stem cell therapy for neurological diseases: dreams and reality Nat Rev Neurosci 2002 3 401 409 10.1038/nrn809 11988779

[B15] Rosser AE Zietlow R Dunnett SB Stem cell transplantation for neurodegenerative diseases Curr Opin Neurol 2007 20 688 692 10.1097/WCO.0b013e3282f132fc 17992090

[B16] Klein SM Behrstock S McHugh J Hoffmann K Wallace K Suzuki M Aebischer P Svendsen CN GDNF delivery using human neural progenitor cells in a rat model of ALS Hum Gene Ther 2005 16 509 521 10.1089/hum.2005.16.509 15871682

[B17] Suzuki M McHugh J Tork C Shelley B Klein SM Aebischer P Svendsen CN GDNF secreting human neural progenitor cells protect dying motor neurons, but not their projection to muscle, in a rat model of familial ALS PLoS One 2007 2 e689 10.1371/journal.pone.0000689 17668067 PMC1925150

[B18] Behrstock S Ebert A McHugh J Vosberg S Moore J Schneider B Capowski E Hei D Kordower J Aebischer P Svendsen CN Human neural progenitors deliver glial cell line-derived neurotrophic factor to parkinsonian rodents and aged primates Gene Ther 2006 13 379 388 10.1038/sj.gt.3302679 16355116

[B19] Alvarez CV Garcia-Lavandeira M Garcia-Rendueles ME Diaz-Rodriguez E Garcia-Rendueles AR Perez-Romero S Vila TV Rodrigues JS Lear PV Bravo SB Defining stem cell types: understanding the therapeutic potential of ESCs, ASCs, and iPS cells J Mol Endocrinol 2012 49 R89 R111 10.1530/JME-12-0072 22822049

[B20] Tsukamoto A Uchida N Capela A Gorba T Huhn S Clinical translation of human neural stem cells Stem Cell Res Ther 2013 4 102 10.1186/scrt313 23987648 PMC3854682

[B21] Jang J Yoo JE Lee JA Lee DR Kim JY Huh YJ Kim DS Park CY Hwang DY Kim HS Kang HC Kim DW Disease-specific induced pluripotent stem cells: a platform for human disease modeling and drug discovery Exp Mol Med 2012 44 202 213 10.3858/emm.2012.44.3.015 22179105 PMC3317484

[B22] Takahashi K Yamanaka S Induction of pluripotent stem cells from mouse embryonic and adult fibroblast cultures by defined factors Cell 2006 126 663 676 10.1016/j.cell.2006.07.024 16904174

[B23] Karumbayaram S Lee P Azghadi SF Cooper AR Patterson M Kohn DB Pyle A Clark A Byrne J Zack JA Plath K Lowry WE From skin biopsy to neurons through a pluripotent intermediate under Good Manufacturing Practice protocols Stem Cells Transl Med 2012 1 36 43 10.5966/sctm.2011-0001 23197638 PMC3727693

[B24] Dimos JT Rodolfa KT Niakan KK Weisenthal LM Mitsumoto H Chung W Croft GF Saphier G Leibel R Goland R Wichterle H Henderson CE Eggan K Induced pluripotent stem cells generated from patients with ALS can be differentiated into motor neurons Science 2008 321 1218 1221 10.1126/science.1158799 18669821

[B25] Corti S Nizzardo M Simone C Falcone M Nardini M Ronchi D Donadoni C Salani S Riboldi G Magri F Menozzi G Bonaglia C Rizzo F Bresolin N Comi GP Genetic correction of human induced pluripotent stem cells from patients with spinal muscular atrophy Sci Transl Med 2012 4 165ra162 23253609 10.1126/scitranslmed.3004108 PMC4722730

[B26] Jessell TM Neuronal specification in the spinal cord: inductive signals and transcriptional codes Nat Rev Genet 2000 1 20 29 10.1038/35049541 11262869

[B27] Wichterle H Lieberam I Porter JA Jessell TM Directed differentiation of embryonic stem cells into motor neurons Cell 2002 110 385 397 10.1016/S0092-8674(02)00835-8 12176325

[B28] Bragdon B Moseychuk O Saldanha S King D Julian J Nohe A Bone morphogenetic proteins: a critical review Cell Signal 2011 23 609 620 10.1016/j.cellsig.2010.10.003 20959140

[B29] Reinhardt P Glatza M Hemmer K Tsytsyura Y Thiel CS Höing S Moritz S Parga JA Wagner L Bruder JM Wu G Schmid B Röpke A Klingauf J Schwamborn JC Gasser T Schöler HR Sterneckert J Derivation and expansion using only small molecules of human neural progenitors for neurodegenerative disease modeling PLoS One 2013 8 e59252 10.1371/journal.pone.0059252 23533608 PMC3606479

[B30] Muhr J Graziano E Wilson S Jessell TM Edlund T Convergent inductive signals specify midbrain, hindbrain, and spinal cord identity in gastrula stage chick embryos Neuron 1999 23 689 702 10.1016/S0896-6273(01)80028-3 10482236

[B31] Storey KG Goriely A Sargent CM Brown JM Burns HD Abud HM Heath JK Early posterior neural tissue is induced by FGF in the chick embryo Development 1998 125 473 484 9425142 10.1242/dev.125.3.473

[B32] Liu JP Laufer E Jessell TM Assigning the positional identity of spinal motor neurons: rostrocaudal patterning of Hox-c expression by FGFs, Gdf11, and retinoids Neuron 2001 32 997 1012 10.1016/S0896-6273(01)00544-X 11754833

[B33] Echelard Y Epstein DJ St-Jacques B Shen L Mohler J McMahon JA McMahon AP Sonic hedgehog, a member of a family of putative signaling molecules, is implicated in the regulation of CNS polarity Cell 1993 75 1417 1430 10.1016/0092-8674(93)90627-3 7916661

[B34] Liem KF Jr Tremml G Roelink H Jessell TM Dorsal differentiation of neural plate cells induced by BMP-mediated signals from epidermal ectoderm Cell 1995 82 969 979 10.1016/0092-8674(95)90276-7 7553857

[B35] Ulloa F Marti E Wnt won the war: antagonistic role of Wnt over Shh controls dorso-ventral patterning of the vertebrate neural tube Dev Dyn 2010 239 69 76 19681160 10.1002/dvdy.22058

[B36] Zou Y Wnt signaling in axon guidance Trends Neurosci 2004 27 528 532 10.1016/j.tins.2004.06.015 15331234

[B37] Takazawa T Croft GF Amoroso MW Studer L Wichterle H Macdermott AB Maturation of spinal motor neurons derived from human embryonic stem cells PLoS One 2012 7 e40154 10.1371/journal.pone.0040154 22802953 PMC3388990

[B38] Chambers SM Fasano CA Papapetrou EP Tomishima M Sadelain M Studer L Highly efficient neural conversion of human ES and iPS cells by dual inhibition of SMAD signaling Nat Biotechnol 2009 27 275 280 10.1038/nbt.1529 19252484 PMC2756723

[B39] Amoroso MW Croft GF Williams DJ O’Keeffe S Carrasco MA Davis AR Roybon L Oakley DH Maniatis T Henderson CE Wichterle H Accelerated high-yield generation of limb-innervating motor neurons from human stem cells J Neurosci 2013 33 574 586 10.1523/JNEUROSCI.0906-12.2013 23303937 PMC3711539

[B40] Boulting GL Kiskinis E Croft GF Amoroso MW Oakley DH Wainger BJ Williams DJ Kahler DJ Yamaki M Davidow L Rodolfa CT Dimos JT Mikkilineni S MacDermott AB Woolf CJ Henderson CE Wichterle H Eggan K A functionally characterized test set of human induced pluripotent stem cells Nat Biotechnol 2011 29 279 286 10.1038/nbt.1783 21293464 PMC3229307

[B41] Burkhardt MF Martinez FJ Wright S Ramos C Volfson D Mason M Garnes J Dang V Lievers J Shoukat-Mumtaz U Martinez R Gai H Blake R Vaisberg E Grskovic M Johnson C Irion S Bright J Cooper B Nguyen L Griswold-Prenner I Javaherian A A cellular model for sporadic ALS using patient-derived induced pluripotent stem cells Mol Cell Neurosci 2013 56 355 364 23891805 10.1016/j.mcn.2013.07.007 PMC4772428

[B42] Hester ME Murtha MJ Song S Rao M Miranda CJ Meyer K Tian J Boulting G Schaffer DV Zhu MX Pfaff SL Gage FH Kaspar BK Rapid and efficient generation of functional motor neurons from human pluripotent stem cells using gene delivered transcription factor codes Mol Ther 2011 19 1905 1912 10.1038/mt.2011.135 21772256 PMC3188742

[B43] Hu BY Zhang SC Differentiation of spinal motor neurons from pluripotent human stem cells Nat Protoc 2009 4 1295 1304 10.1038/nprot.2009.127 19696748 PMC2789120

[B44] Karumbayaram S Novitch BG Patterson M Umbach JA Richter L Lindgren A Conway AE Clark AT Goldman SA Plath K Wiedau-Pazos M Kornblum HI Lowry WE Directed differentiation of human-induced pluripotent stem cells generates active motor neurons Stem Cells 2009 27 806 811 10.1002/stem.31 19350680 PMC2895909

[B45] Sareen D O’Rourke JG Meera P Muhammad AK Grant S Simpkinson M Bell S Carmona S Ornelas L Sahabian A Gendron T Petrucelli L Baughn M Ravits J Harms MB Rigo F Bennett CF Otis TS Svendsen CN Baloh RH Targeting RNA foci in iPSC-derived motor neurons from ALS patients with a C9ORF72 repeat expansion Sci Transl Med 2013 5 208ra149 10.1126/scitranslmed.3007529 24154603 PMC4090945

[B46] Wada T Honda M Minami I Tooi N Amagai Y Nakatsuji N Aiba K Highly efficient differentiation and enrichment of spinal motor neurons derived from human and monkey embryonic stem cells PLoS One 2009 4 e6722 10.1371/journal.pone.0006722 19701462 PMC2726947

[B47] Zeng H Guo M Martins-Taylor K Wang X Zhang Z Park JW Zhan S Kronenberg MS Lichtler A Liu HX Chen FP Yue L Li XJ Xu RH Specification of region-specific neurons including forebrain glutamatergic neurons from human induced pluripotent stem cells PLoS One 2010 5 e11853 10.1371/journal.pone.0011853 20686615 PMC2912324

[B48] Zhang SC Wernig M Duncan ID Brustle O Thomson JA In vitro differentiation of transplantable neural precursors from human embryonic stem cells Nat Biotechnol 2001 19 1129 1133 10.1038/nbt1201-1129 11731781

[B49] Lee H Shamy GA Elkabetz Y Schofield CM Harrsion NL Panagiotakos G Socci ND Tabar V Studer L Directed differentiation and transplantation of human embryonic stem cell-derived motoneurons Stem Cells 2007 25 1931 1939 10.1634/stemcells.2007-0097 17478583

[B50] Erceg S Lainez S Ronaghi M Stojkovic P Perez-Arago MA Moreno-Manzano V Moreno-Palanques R Planells-Cases R Stojkovic M Differentiation of human embryonic stem cells to regional specific neural precursors in chemically defined medium conditions PLoS One 2008 3 e2122 10.1371/journal.pone.0002122 18461168 PMC2346555

[B51] Rungsiwiwut R Manolertthewan C Numchaisrika P Ahnonkitpanit V Virutamasen P Techakumphu M Pruksananonda K The ROCK inhibitor Y-26732 enhances the survival and proliferation of human embryonic stem cell-derived neural progenitor cells upon dissociation Cells Tissues Organs 2013 198 127 138 10.1159/000354031 24158103

[B52] López-González R Camacho-Arroyo I Velasco I Progesterone and 17beta-estradiol increase differentiation of mouse embryonic stem cells to motor neurons IUBMB Life 2011 63 930 939 10.1002/iub.560 21901819

[B53] Xue H Wu S Papadeas ST Spusta S Swistowska AM MacArthur CC Mattson MP Maragakis NJ Capecchi MR Rao MS Zeng X Liu Y A targeted neuroglial reporter line generated by homologous recombination in human embryonic stem cells Stem Cells 2009 27 1836 1846 10.1002/stem.129 19544414 PMC2741170

[B54] Parker GC Li X Anguelov RA Toth G Cristescu A Acsadi G Survival motor neuron protein regulates apoptosis in an in vitro model of spinal muscular atrophy Neurotox Res 2008 13 39 48 10.1007/BF03033366 18367439

[B55] Marchetto MC Muotri AR Mu Y Smith AM Cezar GG Gage FH Non-cell-autonomous effect of human SOD1 G37R astrocytes on motor neurons derived from human embryonic stem cells Cell Stem Cell 2008 3 649 657 10.1016/j.stem.2008.10.001 19041781

[B56] Han SS Williams LA Eggan KC Constructing and deconstructing stem cell models of neurological disease Neuron 2011 70 626 644 10.1016/j.neuron.2011.05.003 21609821

[B57] Jung YW Hysolli E Kim KY Tanaka Y Park IH Human induced pluripotent stem cells and neurodegenerative disease: prospects for novel therapies Curr Opin Neurol 2012 25 125 130 10.1097/WCO.0b013e3283518226 22357218 PMC3786112

[B58] Badger JL Cordero-Llana O Hartfield EM Wade-Martins R Parkinson’s disease in a dish - using stem cells as a molecular tool Neuropharmacology 2014 76 88 96 24035919 10.1016/j.neuropharm.2013.08.035

[B59] Hermann A Storch A Induced neural stem cells (iNSCs) in neurodegenerative diseases J Neural Transm 2013 120 S19 S25 10.1007/s00702-013-1042-9 23720190

[B60] Qu Q Li D Louis KR Li X Yang H Sun Q Crandall SR Tsang S Zhou J Cox CL Cheng J Wang F High-efficiency motor neuron differentiation from human pluripotent stem cells and the function of Islet-1 Nat Commun 2014 5 3449 24622388 10.1038/ncomms4449

